# Skills assessment for laparoscopic surgery based on computer analysis metrics: ScopePro Trainer

**DOI:** 10.1007/s00464-025-12017-4

**Published:** 2025-08-11

**Authors:** Daniel Lorias-Espinoza, Sandra Karina Díaz Cardona, Leobardo Elí Sánchez Velasco, Ricardo Manuel Ordorica-Flores, Arturo Minor-Martínez, Salvador Montoya-Alvarez, Fernando Pérez-Escamirosa

**Affiliations:** 1 Sección de Bioelectrónica, Departamento de Ingeniería Eléctrica, Centro de Investigación y de Estudios Avanzados del Instituto Politécnico Nacional (CINVESTAV–IPN), Ciudad de Mexico, Mexico; 2 Servicio de Cirugía Endoscópica, Hospital Infantil de México Federico Gómez, Calle Dr. Márquez No. 162, Cuauhtémoc, Doctores, 06720 Ciudad de Mexico, Mexico; 3 Instituto de Ciencias Aplicadas y Tecnología (ICAT), Universidad Nacional Autónoma de México (UNAM), Circuito Exterior S/N, Ciudad Universitaria, Coyoacán, 04510 Ciudad de Mexico, Mexico

**Keywords:** Objective assessment, Surgical training, Computer vision, ScopePro, Validation

## Abstract

**Background:**

The aim of this study is to present the construct validity of the ScopePro Trainer system and determine its effectiveness as a training and objective assessment tool for surgeons’ psychomotor laparoscopic skills.

**Methods:**

Four surgeons, eight PG2 to PG4 residents, and twenty medical students participated in this study. All participants performed three FLS-based tasks using conventional laparoscopic instruments and the ScopePro Trainer system. Using the linearity principle of the spiral, a passive marker was added to each instrument, shaped like a spiral. Six metrics related to time, linear motion, and angular motion were used to assess the laparoscopic performance of the participants in all tasks. Statistical analysis was performed using the analysis of variance (ANOVA) test, followed by a Student’s *t* test with Tukey’s correction for multiple testing.

**Results:**

The performance of ScopePro Trainer is illustrated by the graphical representation of the instruments' paths and the calculations of metrics derived from their position and rotation. Outcomes presented significant differences in the participant's skills during the execution of three surgical training tasks.

**Conclusions:**

The ScopePro Trainer system has been presented and successfully validated. Outcomes showed that ScopePro was able to differentiate between participants with varying levels of laparoscopic expertise. The ScopePro Trainer offers an alternative to traditional tracking systems for analyzing the rotational motion of surgical instruments.

Minimally invasive surgery (MIS) has many well-proven benefits. As a result, it is being used more and more every day and is being adopted by various surgical specialties. However, it requires the surgeon to develop a range of skills. The laparoscopic surgeon-in-training must develop a series of knowledge and complex non-innate skills, such as hand–eye coordination, depth perception, and psychomotor skills, in addition to those related to the surgical procedure. One of the several skills that the surgeon must develop is the efficient handling of surgical instruments.

Laparoscopic simulations have helped trainees learn basic surgical skills in the laboratory while reserving the operating room for the training of advanced skills, highlighting the importance of technological developments in conjunction with didactic and evaluative approaches in medical–surgical training [1, 2]. There are two prominent types of laparoscopic simulators: virtual simulators and physical simulators (commonly referred to as Box trainers). Virtual simulators offer immersive learning experiences through the integration of virtual and augmented reality. These simulators typically feature multiple built-in sensor systems to capture various metrics, enabling the objective evaluation of laparoscopic skills. However, the lack of real instruments presents a challenge in recreating realistic haptics, and the cost of these systems is usually high. Box trainers, on the other hand, focus on developing psychomotor skills using real instruments. Although these systems typically provide less information than their virtual counterparts, most box trainers still offer sufficient data to evaluate users objectively and tend to be more affordable systems.

A frequently used method by simulators to evaluate surgical proficiency is motion analysis. There are different technological options for the recording and evaluation of laparoscopic surgical movements, for example, the proposal based on video analysis for the evaluation of laparoscopic psychomotor skills, EVA [3] by Oropesa et al., and the proposal of software for the evaluation of surgical skills by Ganni et al. [4]. These options analyze the execution of a laparoscopic procedure offline and use software to identify, process, and determine the position of the instruments, subsequently generating evaluation metrics. Furthermore, the motion analysis systems can be classified as passive and active. Passive systems consist of a video-based tracking system with markers attached to the instruments [5, 6]. These markers have different geometric shapes and can be labels of different colors. Each marker has a distinct setting and is used only for a specific instrument [7]. On the other hand, active systems are characterized by the fact that the sensor requires an electrical power source, which is usually supplied via batteries or cables connected to a power source. These sensors can either add weight or restrict the natural movement of the surgeon’s hand. An example of these active systems is the proposal by Heiliger et al. [8], which features an active tracking system based on inertial sensors. Most of the mentioned systems measure linear motion; however, from a technological point of view, very few systems allow the recording of the instrument's angular motion, such as rotation around its axis. In addition, methodologically, not all evaluation methods take this movement into account.

The objective of this study is to present the ScopePro Trainer system and evaluate construct validity. ScopePro is a low-cost, portable laparoscopic box simulator equipped with an optical recording system utilizing passive markers to measure the rotation of the laparoscopic instrument tip along its traditional linear motion. This training system offers a viable alternative to surgical training programs and demonstrates its potential as a valuable tool for the acquisition and objective assessment of laparoscopic skills.

## Materials and methods

The present study validates the ScopePro Trainer system and assesses the reliability of six motion metrics used for analyzing spiral markers in three skill tasks. Our proposal is designed and developed for education, training, and valuation of the surgeon’s psychomotor skills in laparoscopic surgery. This study was conducted at the Pediatric Surgery Service of Hospital Infantil de México Federico Gómez in Mexico City, Mexico.

### ScopePro trainer system

This study utilized a previously reported laparoscopic box trainer, which provides visual feedback via a 10 mm USB camera that emulates a zero-degree laparoscope and features uniform illumination with cool white light. The ports are arranged in a diamond shape, allowing for different locations of the laparoscopic instruments [9]. The dimensions of the trainer are 21 cm high, 28 cm wide, and 40 cm deep. The box trainer consists of a pair of webcams installed inside the trainer, which together with an algorithm registers the position and rotation of instruments for each hand. The measurement is performed in both hands while completing three laparoscopic tasks from the FLS training protocol.

### Calculation of the three-dimensional position of the instruments

Programming was done using Python. The steps of the application were as follows: first, images are acquired. Subsequently, a blur filter is applied to smooth the image and improve the color detection by reducing noise. After smoothing, the image is converted from RGB color space to HSV using the cvtColor function of the OpenCV library. H, S, and V-value filters are applied to previously selected colors (The instrument markers colors), and a mask is applied to remove all parts of the image that do not contain the selected color. Once the masks were obtained, two pairs of morphological operations (erosion and dilation) were applied to remove unwanted selections. After morphological operations are applied, the masks contain only the areas of the selected color.

The findContours function highlights all the outlines of the detected areas. Subsequently, the distortion (tangential and radial) of the lens is corrected by applying the undistortPoints function of the OpenCV library. Finally, the function returns the newly corrected coordinates for calculating the three-dimensional position. The three-dimensional error of the instrument position was calculated to be ± 2 mm.

### Instrument rotation calculation

**Fig. 1:**
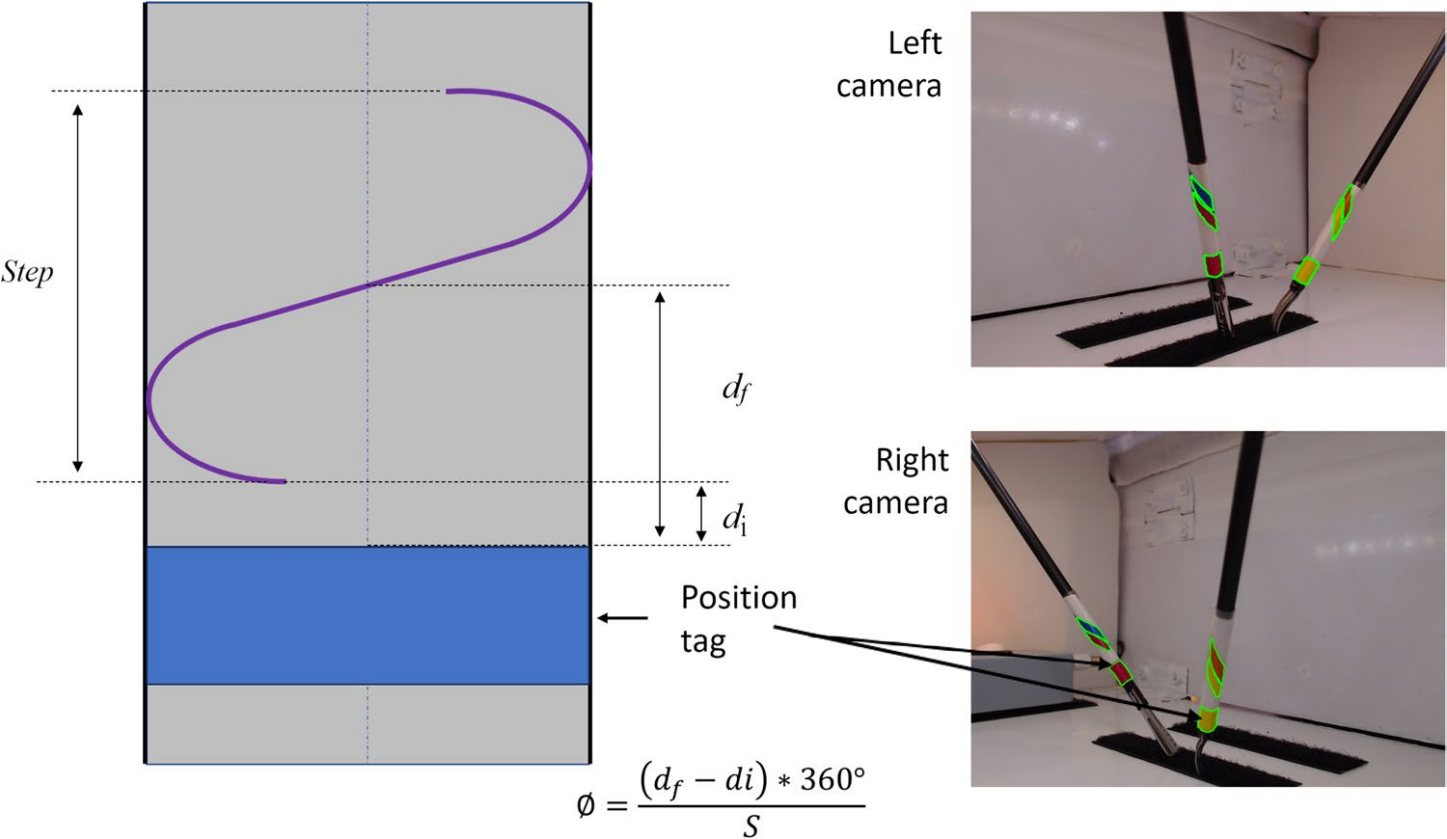
Representation of the different markers (spiral marker and placeholder) in the distal part of the laparoscopic instruments. ${d}_{f}$ is the distance from the instrument placeholder to the point where the coil coincides with the central axis of the instrument. ${d}_{i}$ is the distance between the predetermined point and the start of the turn. *Step* (*S*) is the helix pitch. The right shows the processing of the images by detecting the contours of the labels

The Archimedean spiral has a geometrical relationship between the rotation angle $\theta$ and the distance traveled by the cylindrical spiral. In other words, it is the conversion of the “step of a spiral” into a rotation angle $\theta$. The angle $\theta$ represents the amount of rotation measured in degrees, where 360 degrees is equivalent to a full rotation. Due to the geometry of the spiral, the height traveled from the base or beginning of the spiral to its current position is proportional to the rotation angle $\theta$. Taking advantage of the geometrical principle that, between “step and step” of a spiral, the distance is constant, the distance ($d$) from the beginning of the spiral to a point in the spiral will also be proportional to the rotation angle $\theta$ (Fig. 1).

### Participants

In this study, medical students, residents in training, and experienced surgeons from the Hospital Infantil de México Federico Gómez were invited to participate. The group of experts (E) consisted of four pediatric surgeons who had performed more than 100 laparoscopic procedures. The intermediate group (I) consisted of eight pediatric surgery residents in training, ranging from PGY-2 to PGY-4, who had performed fewer than 10 laparoscopic surgical procedures. The novice group (N) consisted of 20 medical students in their final year of education, with no prior experience in laparoscopic surgery, all from the Unidad de Simulación de Posgrado (USIP) of the Faculty of Medicine of the Universidad Nacional Autonoma de México (UNAM). Before starting the study, written informed consent was obtained from each participant. The research protocol for this study was approved by the Institutional Review Board (IRB) of the Hospital Infantil de México Federico Gómez under research number HIM-AE 2023-005.

### Validation

**Fig. 2:**
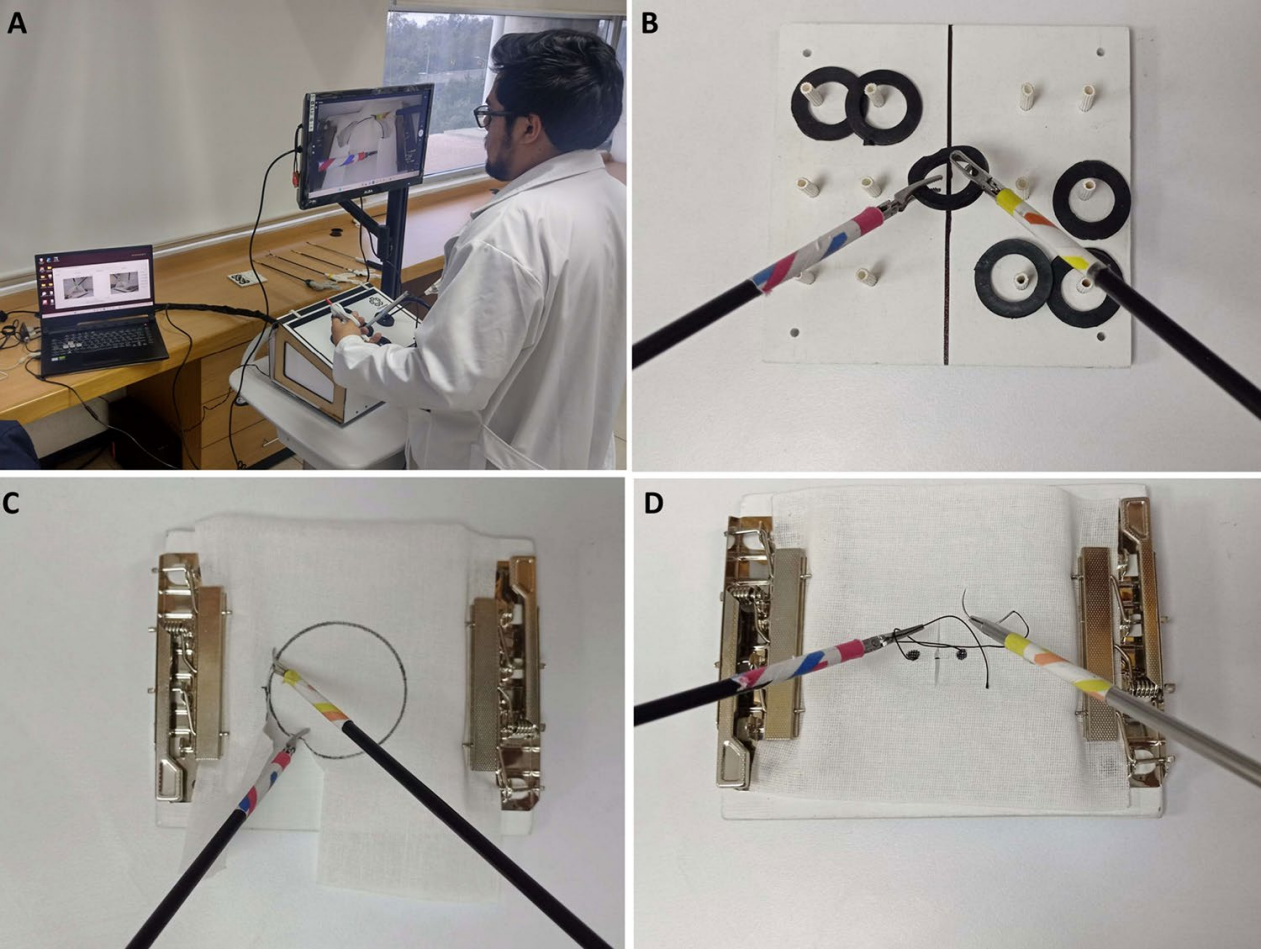
**A** Setup of the training system developed at CINVESTAV–IPN. On the laptop, it features the evaluation system, and on the TV screen, it provides visual feedback for the surgeons. **B** Peg transfer, **C** Pattern cutting, and **D** Intracorporeal knots tasks

To evaluate the construct validity of the ScopePro Trainer system, all participants performed a series of three laparoscopic tasks (Fig. 2). All participants provided written informed consents and agreed to the processing of their data.

- Peg transfer: The task was to transfer six rubber rings from the left side of a board to the right side of the board. The rings are grasped with the right hand and transferred to the left hand in the air using the laparoscopic grasper (Fig. 2B) when transitioning from right to left and vice versa when transitioning from left to right. This task involves bimanual manipulation skills, grasping, hand–eye coordination, and spatial perception [10].
- Pattern cutting: Participants cut a 4.5 cm diameter circular pattern marked into a 13 × 13 cm piece of fabric stretched on a plastic base (Fig. 2C). Using the laparoscopic scissors in their dominant hand, the participant cut the drawn circle as closely as possible to the line. The task was finished when the circle was completely cut and separated from the fabric. This exercise required skills in cutting, grasping, precision, and hand–eye coordination [10].
- Intracorporeal knots: The tasks consisted of grasping the suture needle with the laparoscopic needle driver, puncturing, and knotting a 12-cm long suture through two predefined points in a piece of fabric measuring 13 × 13 cm (Fig. 2D). The suture was tied using an intracorporeal knot technique. For all trials, a 2–0 silk suture on a 26-mm taper needle was used. This task involved skills at needle manipulation, management of silk suture, knot tying, and bimanual dexterity [10].

For all three tasks, the participant, at the beginning of each repetition, placed the instruments in a home plate position, which consists of bringing the tips of both instruments together inside the trainer. At the end of the task, the instruments are returned to their home position.

### Performance parameters

For all three tasks, data were evaluated according to laparoscopic experience using the metrics shown in Table 1. MATLAB Release 2019b (MathWorks, Natick, Massachusetts, USA) was used to display the trajectories of markers placed on the instruments and calculate motion-related metrics.

**Table 1:** Parameters for the analysis of laparoscopic psychomotor skills based on the rotation of the instruments on their own axis

Metric	Definition	Equation
Time	Total execution time of the task. Units in seconds (seconds)	$T=n\Delta t$
Path length (PL)	Total path followed by the tip of the instrument while performing the task(s). (Meters)	$\text{PL}=\sum_{k=0}^{n-2}\sqrt{{\left(x\left(k+1\right)-x\left(k\right)\right)}^{2}+{\left(y\left(k+1\right)-y\left(k\right)\right)}^{2}+{\left(z\left(k+1\right)-z\left(k\right)\right)}^{2}}$
Clockwise turns (N_clk_)	Number of turns performed by the instrument clockwise. (revolutions)	${N}_{\text{clk}}= \sum_{k=0}^{n-2}\frac{\left|\phi \left(k+1\right)-\phi \left(k\right)\right|}{360^\circ }[\phi \left(k+1\right)-\phi \left(k\right)>0]$
Counterclockwise turns (N_cclk_)	Number of turns performed by the instrument counterclockwise. (revolutions)	${N}_{\text{cclk}}=\sum_{k=0}^{n-2}\frac{\left|\phi \left(k+1\right)-\phi \left(k\right)\right|}{360^\circ }[\phi \left(k+1\right)-\phi \left(k\right)<0]$
Number of turns (NT)	Total number of turns performed by the instrument. (revolutions)	$\text{NT}={N}_{\text{clk}}+{N}_{\text{cclk}}$
Ratio (R)	The relationship between instrument rotation (NT) and total path length. (revolutions/meter)	$R=\frac{{N}_{\text{clk}}+{N}_{\text{cclk}}}{\text{PL}}$

*n* represents the number of samples acquired in a task $[\text{Adimentional}]$. $\Delta t$ represent the sampling period of the measurement system $[\text{seconds}]$

$x\left(k\right)$, $y\left(k\right)$, $z\left(k\right)$ represent the measured instrument tip position in the 3-dimensional space in axis *X*, *Y*, and *Z*, respectively at the *k* sample $[\text{meters}]$

$\phi \left(k\right)$ represents the spiral angle at the k sample $[\text{Degrees}\bullet \text{Revolution}]$. $[\phi \left(k+1\right)-\phi \left(k\right)>0]$ and $[\phi \left(k+1\right)-\phi \left(k\right)<0]$ are Iverson Brackets which evaluate to 1 if the condition inside is true, or 0 if the condition inside es false

### Statistical analysis

Statistical analysis was performed using R version 3.6 for Windows. For validation, parametric tests were used to compare groups in this study. Analysis of Variance (ANOVA) test was used to identify statistically significant differences in performance between the groups. In contrast, Student’s *t* test with the Tukey’s correction for multiple testing was used for pairwise group comparisons. A level of *p* ≤ 0.05 was considered statistically significant.

## Results

A total of 32 participants were included: 20 Novices (mean age 24.18 ± 2.09 years, 14 right-hand dominant and 6 left-hand dominant), 8 Intermediates (mean age 32.20 ± 1.92 years, 7 right-hand dominant and 1 left-hand dominant), and 4 experts (mean age 47.50 ± 8.89 years, 4 right-hand dominant and 0 left-hand dominant). All participants performed two repetitions of each task, and the average of both performances was used for statistical analysis.

For the three levels of experience, the instrument’s movement of each hand during task execution was graphically recorded. These were automatically generated and calculated offline upon completion of all tasks for each participant. In addition, the rotation of the instruments was also recorded. From these records, the six metrics for each of the three FLS tasks were analyzed, related to the time, motion, and turning of the instruments.

Tables 2, 3, and 4 report the mean and standard deviation for each analyzed metric, along with the *p* values obtained from hypothesis testing and the corresponding 95% confidence intervals for peg transfer, pattern-cutting, and intracorporeal knots tasks, respectively.

**Table 2:** Performance results and hypothesis testing for the peg transfer task

Metrics	Nov Mean (SD)	Int Mean (SD)	Exp Mean (SD)	N–I–E^b^ *p* value	I–E^a^ *p* value (Conf Int)	N–E^a^ *p* value (Conf Int)	N–I^a^ *p* value (Conf Int)
Time (s)	304.46 (142.32)	192.87 (48.32)	187.56 (22.67)	0.107	0.997 (− 187.52, 198.13)	0.204 (− 50.93, 284.73)	0.185 (− 43.44, 266.63)
*Right hand*							
Path length (m)	11.28 (7.25)	5.57 (1.60)	3.99 (1.16)	0.062	0.909 (− 8.12, 11.28)	0.098 (− 1.16, 15.73)	0.176 (− 2.10, 13.51)
Clockwise turns (rev)	15.61 (7.38)	14.94 (2.00)	13.94 (1.14)	0.883	0.963 (− 8.92, 10.92)	0.874 (− 6.97, 10.30)	0.975 (− 7.31, 8.64)
Counterclockwise turns (rev)	8.99 (7.42)	0.038 (0.03)	0.32 (0.64)	**0.011**	0.997 (− 10.09, 9.53)	**0.046** **(0.13, 17.21)**	**0.025** **(1.06, 16.84)**
Total turns (rev)	23.37 (9.48)	14.98 (1.99)	14.26 (1.62)	0.054	0.988 (− 11.95, 13.40)	0.116 (− 1.92, 20.15)	0.117 (− 1.81, 18.58)
Ratio (rev/m)	3.82 (3.78)	2.94 (0.75)	3.86 (0.79)	0.844	0.889 (− 5.98, 4.15)	1.000 (− 4.44, 4.37)	0.846 (− 3.19, 4.95)
*Left hand*							
Path length (m)	7.49 (4.04)	4.54 (1.08)	3.72 (1.03)	0.090	0.921 (− 4.64, 6.29)	0.134 (− 0.98, 8.53)	0.225 (− 1.44, 7.34)
Clockwise turns (rev)	17.74 (15.31)	25.31 (8.11)	22.49 (5.95)	0.523	0.941 (− 18.92, 24.57)	0.798 (− 23.68, 14.18)	0.520 (− 25.06, 9.91)
Counterclockwise turns (rev)	10.16 (6.20)	6.63 (2.86)	5.44 (3.54)	0.233	0.937 (− 7.70, 10.09)	0.286 (− 3.01, 12.47)	0.432 (− 3.62, 10.69)
Total turns (rev)	26.67 (15.01)	31.95 (7.01)	27.93 (3.16)	0.724	0.874 (− 16.77, 24.81)	0.983 (− 19.35, 16.84)	0.702 (− 21.99, 11.44)
Ratio (rev/m)	5.36 (4.10)	1.47 (0.65)	8 (2.28)	**0.026**	**0.023** **(−** **12.21, -0.84)**	0.379 (− 7.58, 2.31)	0.102 (− 0.67, 8,46)

^a^Student’s *t* test with Tukey’s correction for differences between pair of groups; significant at *p* < 0.05 (bold)

^b^ANOVA test for differences across the three groups; significant at *p* < 0.05 (bold)

**Table 3:** Performance results and hypothesis testing for the pattern-cutting task

Metrics	Nov Mean (SD)	Int Mean (SD)	Exp Mean (SD)	N–I–E^b^ *p* value	I–E^a^ *p* value (Conf Int)	N–E^a^ *p* value (Conf Int)	N–I *p* value (Conf Int)
Time (s)	488.50 (231.93)	470.03 (405.48)	266.66 (38.97)	0.366	0.503 (− 253.87, 660.61)	0.348 (− 176.13, 619.82)	0.991 (− 349.16, 386.11)
*Right hand*							
Path length (m)	19.31 (8.42)	9.29 (2,31)	7.5 (1.53)	**0.007**	0.914 (− 9.55, 13.13)	**0.018** **(1.94, 21.67)**	**0.030** **(0.90, 19.13)**
Clockwise turns (rev)	16.38 (9.44)	13.53 (4.08)	9.75 (4.33)	0.351	0.750 (− 9.52, 17.07)	0.330 (− 4.95, 18.20)	0.776 (− 7.84, 13.54)
Counterclockwise turns (rev)	9.60 (7.07)	1.33 (1.27)	3.97 (4.40)	**0.038**	0.776 (− 12.55, 7.27)	0.244 (− 3.00, 14.25)	**0.041** **(0.29, 16.24)**
Total turns (rev)	24.75 (13.65)	14.86 (4.67)	13.73 (1.71)	0.127	0.986 (− 17.34, 19.60)	0.213 (− 5.06, 27.09)	0.231 (− 4.96, 24.74)
Ratio (rev/m)	2.58 (3.74)	1.68 (0.36)	1.83 (0.25)	0.813	0.996 (− 5.11, 4.80)	0.898 (− 3.57, 5.06)	0.832 (− 3.08, 4.89)
*Left hand*							
Path length (m)	9.62 (4.07)	4.96 (1.50)	4.09 (1.62)	**0.011**	0.917 (− 4.76, 6.51)	**0.026** **(0.63, 10.44)**	**0.043** **(0.13, 9.19)**
Clockwise turns (rev)	19.50 (12.17)	34.07 (12.87)	32.18 (8.78)	0.058	0.969 (− 18.45, 22.23)	0.188 (− 30.38, 5.03)	0.085 (− 30.92, 1.79)
Counterclockwise turns (rev)	11.48 (8.87)	5.52 (2.02)	3.86 (2.48)	0.125	0.933 (− 10.31, 13.62)	0.176 (− 2.80, 18.03)	0.277 (− 3.66, 15.59)
Total turns (rev)	29.76 (18.44)	39.59 (12.26)	36.04 (10.46)	0.502	0.941 (− 23.91, 31.01)	0.781 (− 30.19, 17.62)	0.502 (− 31.92, 12.24)
Ratio (rev/m)	5.22 (4.10)	8.34 (2.03)	9.19 (2.79)	0.108	0.930 (− 6.87, 5.16)	0.157 (− 9.21, 1.27)	0.251 (− 7.96, 1.72)

^a^Student’s *t* test with Tukey’s correction for differences between pair of groups; significant at *p* < 0.05 (bold)

^b^ANOVA test for differences across the three groups; significant at *p* < 0.05 (bold)

**Table 4:** Performance results and hypothesis testing for the intracorporeal knots task

Metrics	Nov Mean (SD)	Int Mean (SD)	Exp Mean (SD)	N–I–E^b^ *p* value	I–E^a^ *p* value (Conf Int)	N–E^a^ *p* value (Conf Int)	N–I *p* value (Conf Int)
Time (s)	449.68 (202.31)	259.6 (41.24)	175.17 (60.54)	**0.015**	0.711 (− 188.35, 357.20)	**0.022** **(37.08, 511.92)**	0.096 (− 29.24, 409.393)
*Right hand*							
Path length (m)	15.66 (12.53)	7.10 (3.13)	4.50 (2.54)	0.110	0.917 (− 14.23, 19.44)	0.154 (− 3.50, 25.81)	0.264 (− 4.98, 22.09)
Clockwise turns (rev)	28.28 (11.70)	18.83 (4.60)	12.4 (4.88)	**0.024**	0.579 (− 9.87, 22.73)	**0.027** **(1.69, 30.07)**	0.184 (− 3.65, 22.55)
Counterclockwise turns (rev)	1.98 (3.97)	0.01 (0.01)	0.01 (0.01)	0.378	1.000 (− 5.23, 5.24)	0.523 (− 2.59, 6.52)	0.471 (− 2.25, 6.17)
Total turns (rev)	29.03 (11.87)	18.85 (4.59)	12.41 (4.89)	**0.019**	0.587 (− 10.08, 22.95)	**0.022** **(2.25, 30.99)**	0.151 (− 3.09, 23.46)
Ratio (rev/m)	3.74 (3.52)	3.00 (1.11)	3.29 (1.01)	0.879	0.987 (− 5.08, 4.51)	0.959 (− 3.72, 4.63)	0.877 (− 3.12, 4.59)
*Left hand*							
Path length (m)	10.53 (9.62)	6.17 (2.43)	3.79 (2.38)	0.276	0.886 (− 10.59, 15.35)	0.301 (− 4.54, 18.03)	0.543 (− 6.07, 14.79)
Clockwise turns (rev)	58.31 (39.92)	32.77 (7.91)	32.82 (15.98)	0.228	1.000 (− 54.39, 54.30)	0.372 (− 21.81, 72.79)	0.316 (− 18.16, 69.23)
Counterclockwise turns (rev)	8.70 (5.33)	6.25 (1.59)	1.61 (0.70)	**0.031**	0.251 (− 2.55, 11.81)	**0.025** **(−** **0.84, 13.34)**	0.532 (− 3.32, 8.23)
Total turns (rev)	65.78 (43.41)	39.02 (9.24)	34.44 (16.09)	0.201	0.978 (− 54.39, 63.55)	0.286 (− 19.98, 82.67)	0.340 (− 20.65, 74.18)
Ratio (rev/m)	10.10 (4.63)	7.95 (3.09)	10.77 (4.49)	0.567	0.599 (− 10.20, 4.56)	0.961 (− 7.10, 5.75)	0.631 (− 3.79, 8.08)

^a^Student’s *t* test with Tukey’s correction for differences between pair of groups; significant at *p* < 0.05 (bold)

^b^ANOVA test for differences across the three groups; significant at *p* < 0.05 (bold)

For the peg transfer task, the metrics that distinguished between the different levels of experience were “Counterclockwise turns” for the right hand and “Ratio” for the left hand. In the pattern-cutting task during the handling of the laparoscopic instrument in the right hand, a lower number of turns were noticeable in the right hand compared to the left hand as indicated by the metric 'Number of turns.' Likewise, the Ratio showed the economy of turn motion between the three study groups. Regarding the rotational economy of the instruments, as measured by the Ratio metric, the left hand developed a better rotational movement economy. The intracorporeal knots task was considered the most difficult task in this study. Regarding quantitative findings, significant differences were observed in 4 of 6 metrics, allowing for the distinction between different experience levels.

In Table 4, it becomes clear that the meaning of all motion metrics consistently decreases as the expert level is reached. As the task increased in difficulty, the metric time was also able to differentiate between different skill levels when comparing between the three groups.

**Fig. 3:**
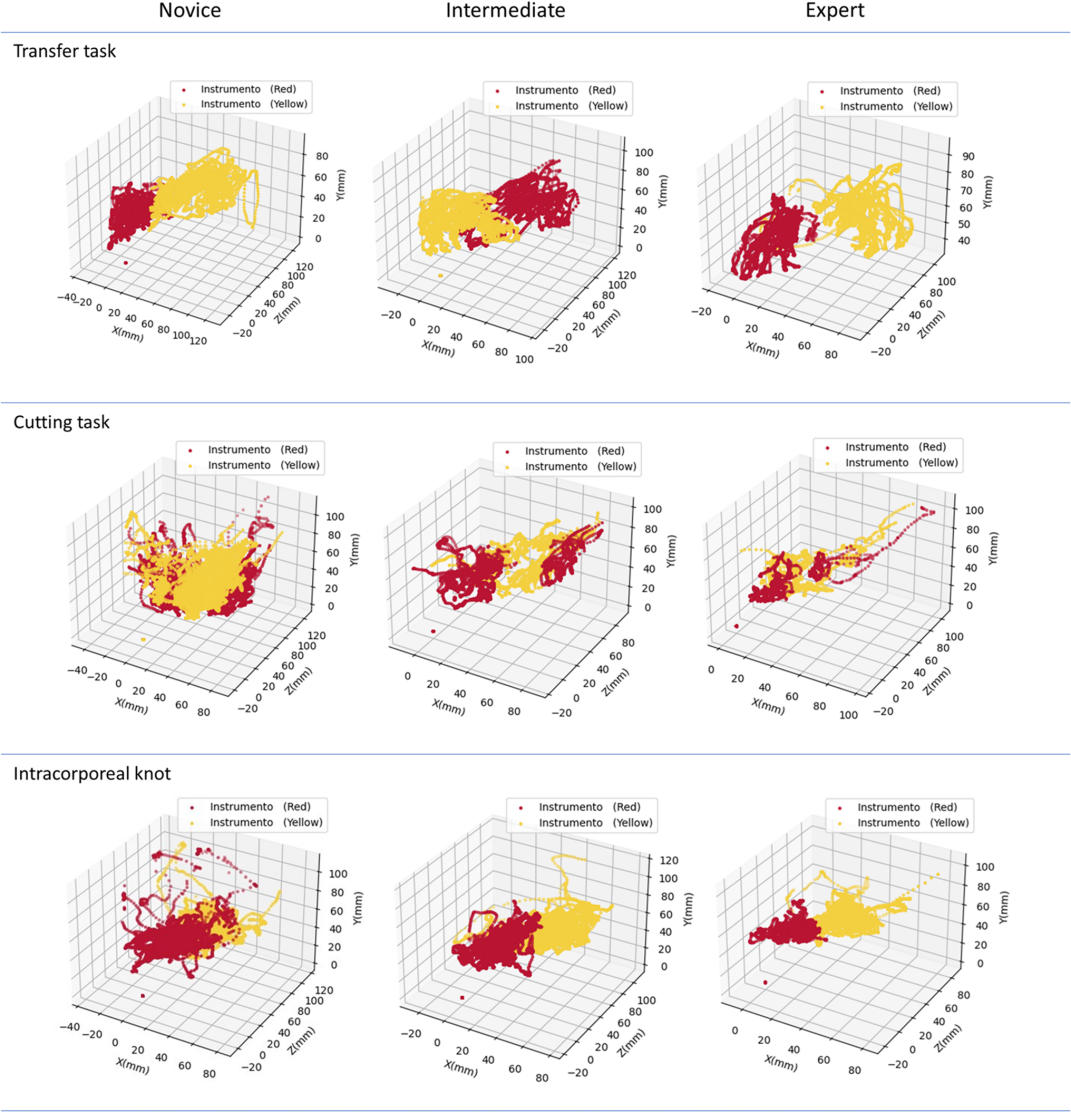
Graphical representation of the trajectories of the instruments in the three laparoscopic tasks for the three levels of experience. The red color represents the laparoscopic instrument in the left hand, and the yellow color represents the laparoscopic instrument in the right hand

This result is associated with the qualitative characteristics of Fig. 3, where a “more compact” pattern of the recorded path of the instruments was generated by the experts. Meanwhile, a less compact trajectory is observed for intermediates, and a scattered and erratic trajectory is observed for novices, representing the greater accuracy of the experts. In the video-based metrics, the number of turns for the right and left hands was a sensitive parameter for evaluating and distinguishing laparoscopic skills in this task by comparing the three different experience levels. Also, path length and time were sensitive enough to differentiate between the three levels of experience and in the pairs of groups. The Ratio in the left hand was able to differentiate in the groups with less dexterity, which has not yet reached ambidexterity.

Comparing the three tasks, the intracorporeal knots task showed the most significant statistical differences, evidencing that the development of psychomotor skills has not yet been achieved. In the pattern-cutting task, the expert surgeons demonstrated significantly better bimanual dexterity, indicating that they made more simultaneous movements of the two instruments compared to the intermediates and novices. Additionally, it was also evident that as the level of dexterity increases, the number of turns tends to decrease. This can be observed in both the right and left hands for the intracorporeal knot task.

## Discussion

It is essential to have indicators, parameters, and metrics that can differentiate between different levels of surgical skill. Laparoscopic surgery is no exception and demands psychomotor dexterity, ambidexterity, hand–eye coordination, adaptation to loss of depth, and, of course, the safe handling of instruments. Laparoscopic surgery is performed using four degrees of freedom. Rotational movements around the axis are essential in laparoscopy, where surgeons must handle instruments with great dexterity to perform procedures in confined spaces and with indirect visualization. Therefore, smooth, controlled, and effective rotations are required.

In the literature, the most evaluated metrics are time, accuracy, errors, and those derived from laparoscopic movement, such as path length and economy of movement. Although metrics derived from motion analysis have proven to be useful for the evaluation of surgical skills, most simulators only analyze linear motion, which represents 3 of the 4 DoF present in laparoscopic settings (movement along the x-axis, y-axis, and z-axis, respectively) and left out the 4th DoF (rotation around the z-axis).

New laparoscopic techniques require complex skills as do new surgical instruments. Addressing the limitations of traditional simulators, such as the lack of feedback on rotational movements, limits the ability to provide objective feedback on movement control in 3D space, which is crucial for improving laparoscopic psychomotor skills. The ability to record instrument rotation in laparoscopic procedures is crucial for understanding and improving dexterity in this minimally invasive surgical technique. Recording these movements can provide several valuable insights into laparoscopic skill training as it allows us to gain a comprehensive understanding of the surgeon's motion performance. The ScopePro system was developed to address the lack of angular motion analysis in low-cost Box trainer systems. In general terms, ScopePro can record instrument rotation around the z-axis in laparoscopic procedures, representing a significant advance in surgical skills training.

Recording rotation around the instrument's axis could help surgeons develop complex motor skills. This capability would enable more realistic surgical scenarios that require precise movements in various planes, angles, and directions. By incorporating rotation recording into simulators, training becomes more aligned with the psychomotor development of minimally invasive psychomotor skills. This improvement could enhance the transfer of skills from training to the operating room as it would provide objective feedback on how precision in movements impacts surgery. The ScopePro system would contribute to more effective training, providing objective and personalized feedback that could improve surgeons’ competency in the surgical setting.

Overall, motion metrics (Path length, Clockwise turns, Counterclockwise turns, and Total turns) along with the time metric showed a tendency to decrease their magnitude as the experience level increased. The Ratio metric indicates the correlation between the number of turns and the instrument path, which can be interpreted as the economy of turns. This analysis showed more economy of turns in the dominant hand. The tendency for the ratio metric was to increase as the skill level increased; this is due to the path length in the denominator of the ratio decreasing faster than the total turns in the numerator. Regarding the differences in the ratio for dominant and non-dominant hands, a learning process could explain which mastery of the right dominant hand is first achieved in most cases. Then, as experience increases, the ambidexterity characteristic of experts is acquired. However, future studies will be carried out to test this hypothesis.

The study indicates that different hand movement metrics, such as the number of turns and Ratio, vary across levels of laparoscopic experience, with dominant hand movements improving first and non-dominant hand movements showing more improvement with increased experience. These findings suggest a learning process in which expertise is characterized by greater efficiency and coordination, potentially leading to ambidextrous behavior in experts. Future research is needed to explore this hypothesis further. Notably, both the number of turns and the ratio metrics were able to distinguish between different skill levels in some instances, where the traditional path length associated with linear motion could not.

A recent study conducted by Heiliger et al. [8] provides strong support for the implementation of simulation techniques to enhance learning using an inertial navigation system, which combines multiple sensors. Three parameters stood out, showing performance improvement. One of these parameters, highlighted, was the number of rotations of the instrument around its axis. The ScopePro Trainer could be integrated into surgical training programs through a combination of complementary methods. Its inclusion will depend on the structure of the curriculum, but it could be useful in the initial stages of training, focusing on basic technical skills training. It could also serve as a complementary tool in simulators to improve proficiency in endoscopic procedures without replacing clinical practice. The cost of the ScopePro Trainer may vary depending on factors, such as model, associated services, and scale of implementation.

For this functional prototype, the cost was approximately 500 USD. This cost per unit could be reduced when produced in higher quantities, making this device a low-cost option for institutions in need of Box trainers with surgical skills evaluation capabilities. Additionally, the portability of the ScopePro system, along with its relatively low cost, means that individuals interested in practicing their skills at home could benefit from this system.

Although the ScopePro Trainer system has demonstrated its ability to differentiate between participants with varying skill levels, further studies are necessary to thoroughly explore its capabilities. First, studies with more expert participants are needed. Although a post hoc power analysis indicates that most metrics have above 80% statistical power, having only four expert surgeons is insufficient to build a sufficiently robust database for automatic, objective evaluation by comparing a new user with known surgeons. Future studies with more extensive teams from other centers and laparoscopic surgeons from other specialties are needed to validate the reliability of the system and enforce its role. Furthermore, future studies are necessary to determine whether these new video-based metrics apply to other tasks. There are other aspects of the validation pending, such as face and content validity. Future studies will revolve around using the Messick validity framework to improve confidence in the ScopePro system. Once the system validation has been expanded, it will be tested in longitudinal studies to research the impact of the system on improving the learning curve of aspiring surgeons, as well as to quantify whether the use of the ScopePro system results in fewer operative errors. These studies will help determine if the acquired skills are transferable to the operating room.

The ScopePro Trainer system, based on computer vision techniques, offers a non-invasive solution for tracking and analyzing the rotational movements of laparoscopic instruments along their axis without altering the surgeons' natural performance. Due to the portability offered by the system, the ScopePro Trainer system can be included in surgical training programs to teach future surgeons.

## Conclusions

In the present manuscript, the ScopePro Trainer system is presented and successfully validated. The results of the construct validity demonstrated the capacities of the ScopePro Trainer to differentiate laparoscopic performance between novice, intermediate, and expert surgeon groups. Furthermore, the system qualitatively demonstrated significant statistical differences in video-based metrics between different laparoscopic skill levels. The ScopePro Trainer offers an alternative to traditional tracking systems for analyzing the rotational motion of surgical instruments. Further research will be conducted using new video-based metrics and laparoscopic skill tasks.
